# Prevalence and Factors of Pregnancy Termination Among Reproductive-Aged Women: Evidence from the Bangladesh Demographic and Health Survey

**DOI:** 10.3390/healthcare12212130

**Published:** 2024-10-25

**Authors:** Md. Rabiul Islam, Makfiratur Rahman, Arifa Farzana Tanha, Nusrat Hossain Sheba, S. M. Raysul Haque, Md. Kamran ul Baset, Zenat Zebin Hossain, Mohammad Abbas Gani, J. M. A. Hannan

**Affiliations:** 1 Department of Public Health, School of Pharmacy and Public Health, Independent University, Bangladesh, Dhaka 1229, Bangladesh; makfiraturrahman@gmail.com (M.R.); kamranspph@iub.edu.bd (M.K.u.B.); zenat@iub.edu.bd (Z.Z.H.); 2 Faculty of Medical Studies, Bangladesh University of Professionals, Dhaka 1216, Bangladesh; arifatanha9@gmail.com; 3 Department of Family and Preventive Medicine, Division of Public Health, University of Utah, Salt Lake City, UT 84108, USA; 4 Department of Pharmacy, Jahangirnagar University, Dhaka 1342, Bangladesh; abg@juniv.edu; 5 Department of Pharmacy, School of Pharmacy and Public Health, Independent University, Bangladesh, Dhaka 1229, Bangladesh

**Keywords:** reproductive-aged women, pregnancy termination, abortion, Bangladesh, Demographic and Health Survey, BDHS 2017–2018

## Abstract

Background: Pregnancy termination (PT) is a major public health concern in low-and middle-income countries like Bangladesh. This cross-sectional study aimed to determine the prevalence and factors of PT using the nationally representative Bangladesh Demographic and Health Survey data 2017–2018. Materials and Methods: A weighted population-based sample of 8759 ever-married reproductive-aged women (15–49 years) was included in the study. The outcome variable was PT in any of the following forms: miscarriage, induced abortion, and stillbirth. A univariate analysis for mean, frequency, and percentage and multiple logistical regression were used to determine the factors associated with PT. Results: Around 18% of the women were found to have PT. The mean age of the women in the study was 25.79 years; 65.1% lived in the rural areas, and the majority of them were Muslims. Advanced age of the women (AOR:3.49, *p* = 0.004), residence in the countryside (AOR:0.81, *p* = 0.002), higher education (AOR:0.72, *p* = 0.027), not being a Muslim (AOR:0.74, *p* = 0.010), higher socio-economic status (AOR:1.28, *p* = 0.027), having a job (AOR:1.15, *p* = 0.041), being married at the age of >22 years (AOR:0.71, *p* = 0.036), and using a mobile phone (AOR:1.22, *p* = 0.002) were significant factors of PT. This study did not find any association between PT and contraceptive use. Conclusions: Age, living region, education, religion, wealth index, working status, marital age, and mobile phone use are the determinants of PT. Interventions including these factors need to be made to reduce PT in Bangladeshi women. These findings could be helpful in undertaking further epidemiological studies to understand the actual causes of PT in various rural and urban settings among different socio-demographic groups in Bangladesh.

## 1. Introduction

Unintended pregnancy often leads to pregnancy termination (PT), which impacts the physical health, mental health, and socio-economic status of women [1]. The latest research estimates that between 2015 and 2019, there were 121 million unplanned pregnancies worldwide (or 64 for every 1000 women) [2], and one-third of those pregnancies are terminated by abortion, miscarriage, menstrual regulation, and stillbirth [3].

Although natural or fatal termination of pregnancy is a regular occurrence among women of childbearing age [2,4], it is never an easy decision to terminate a pregnancy, particularly in low-and-middle-income countries (LMICs), where patriarchal societies, restrictive abortion laws, cultural traditions, religious influences, and economic concerns may all influence women’s choices [5,6]. The World Health Organization (WHO) has reported a higher number of pregnancies and maternal deaths (around 90%) in these regions [7]. According to a previous study, LMICs have a higher rate of PT [Afghanistan (19.5%), India (12.0%), Pakistan (33.4%), and Nepal (20.1%)] than higher-income countries [8]. Because of strict anti-abortion laws in several South Asian countries, the unsafe abortion rate is comparatively high in South Asia [9].

Worldwide, 45% of all abortions are deemed unsafe, with 97% occurring in LMICs [7]. Between 2010 and 2014, around 55.7 million abortions were performed globally to terminate pregnancies [10], with 88% of them occurring in developing countries. Most abortions in LMICs usually take place in secret and are carried out in hazardous ways by untrained people, thereby increasing the risk of complications like hemorrhage, infection, uterus perforation, incomplete abortion, death of the mother, and morbidities [7,11,12,13].

In 2015, around 2.62 million pregnancies led to stillbirths around the world, with developed nations accounting for approximately 1.8% and developing countries accounting for the remainder [14], where Bangladesh was ranked 7th, with 83,000 stillbirths [14]. Similarly, in 2014, around 1,194,000 induced abortions [15] and 430,000 menstrual regulations occurred in Bangladesh [16]. Thus, risky PT is one of the factors that increase maternal health problems [17], mortality, and morbidity [18] and is a key public health issue in many developing nations [19] like Bangladesh.

Contemporary studies have found that several factors, notably maternal age, education, birth order [8], maternal media exposure, contraception use, women’s autonomy, birth order, parity, maternal occupation [20,21], marital status [22], and religion [23], contribute to PT. In addition, genetic factors like a child affected with a genetic disease and an abnormal fetus have also been significantly linked to PT [24,25].

In Bangladesh, the termination of life-threatening pregnancies is increasingly a major reproductive health concern [26,27,28,29]. Moreover, the country’s healthcare system faces a lot of challenges [30]. The prevalence of fatal PT has not significantly changed between 1994 and 2018 [28,29]. Bangladesh, primarily being a male-dominated [31] and Muslim society [32], inhibits female mobility and participation in decision-making processes. In particular, when it comes to family planning (i.e., spacing between pregnancies, total number of pregnancies, PT, etc.), a husband’s or partner’s decision affects the pregnancy outcome [31]. It becomes a maze of legal, religious, and moral concerns in the country [32]. When faced with an undesirable pregnancy, women typically rely on informal or traditional means of pregnancy termination, such as homeopathic and allopathic drugs, contraceptive pills, herbal remedies, roots of plants, consuming hot salt water, and so on [31]. Abortion is illegal [32] for any reason other than safeguarding a woman’s life [16] or for menstrual regulation [33]. Accessing services for PT can be challenging for women [31]. Bangladesh has a high rate of risky, unskilled service providers performing pregnancy terminations [16], which could result in mortality [18] and complexity in maternal health [17].

The Sustainable Development Goals (SDGs) place a strong emphasis on the need to attain gender equality; enable all women and girls to have the power to make decisions about their reproductive health; ensure that everyone has access to family planning, information, and education; and incorporate comprehensive reproductive health initiatives into national policies and programs [34,35]. Given the severe effects of pregnancy termination, it is crucial to comprehend its risk factors to assist policy makers in creating focused preventative and intervention measures for lowering the rate of pregnancy termination.

Understanding regional and nation-specific variations would help healthcare professionals and stakeholders to identify possible reasons for the high prevalence of terminated pregnancies and will direct the prioritization of intervention efforts to the most at-risk countries in LMICs. However, to the best of our knowledge, there is no study that has exclusively examined the prevalence and factors associated with PT derived from the most recent nationally representative data of the Bangladesh Demographic and Health Survey 2017–2018 (BDHS 2017–2018).

Thus, this study intends to fill this crucial knowledge gap by carrying out a study based on BDHS 2017–2018 data to determine the prevalence and associated factors of PT in Bangladesh. This study’s findings may aid in the implementation of different initiatives to reduce the number of abortions and maternal fatalities and improve reproductive health.

## 2. Materials and Methods

### 2.1. Data Source and Study Population

The data for this study were obtained from the latest BDHS 2017–2018, which is a nationally representative cross-sectional survey. It was carried out in collaboration with the Bangladesh National Institute for Population Research and Training (NIPORT) in 2017–2018, and data on the different sociodemographic, fertility, nutrition, and health status of the population were collected following a two-stage stratified cluster sampling technique. Details of the survey methodology and strategies are described in the BDHS 2017–2018 survey report [36]. Primarily, a stratified, multi-stage cluster sample from 675 enumeration areas (EAs) (425 in rural areas and 250 in urban areas) was drawn using probability proportional to the size of the EAs. Secondly, a full list of the households was constructed by using a systematic sampling technique from each EA, which was used as a sampling frame. On average, 30 households were picked from each of the EAs. A total of 20,250 households were selected for the survey, and 20,100 reproductive-aged women were interviewed (150 women did not participate in the survey).

The inclusion criteria of the sample were as follows: (a) women aged between 15 and 49 years, (b) women who had at least one pregnancy within the last five years, (c) women who fully completed the survey so that the study could prevent the loss of important data, and (d) women who gave informed consent.

On the contrary, women beyond the age of 15 to 49 years, women who were unable to give informed consent, and women who had not had at least one pregnancy within the last five years were excluded from the study.

### 2.2. Outcome Variable

The outcome variable of the study was PT of reproductive-aged women, which was a categorical variable. In the questionnaire of the BDHS 2017–2018, a single question inquired if they had ever experienced a miscarriage, induced abortion, or ‘menstrual regulation (MR)’ (a term used in Bangladesh to describe legal abortion by clinicians). Respondents who reported having experienced a miscarriage, induced abortion, or stillbirth were asked whether such an event had occurred within the past five years. The outcome variable PT was dichotomized as ‘0’ and ‘1’ for no PT and PT, respectively. The analysis was restricted to PT that occurred in the last five years before the survey.

### 2.3. Explanatory Variables

Per the guidance of the existing literature and based on the availability of the variables, various explanatory variables were considered for the final analysis of the current study. Several factors such as age (continuous), place of residence (rural or urban, categorical), divisions (categorical), educational status (categorical), religion (categorical), wealth index (categorical), present working status (categorical), possession of a self-mobile phone (categorical), age at marriage (continuous), number of antenatal care (ANC) visits (categorical), and contraceptive use status (categorical) of the ever-married women were considered as independent variables.

### 2.4. Data Collection

BDHS 2017–2018 data were collected using a structured questionnaire including six types of questionnaires: (1) the household questionnaire, (2) the women’s questionnaire (completed by ever-married women age 15–49), (3) the biomarker questionnaire, (4) two verbal autopsy questionnaires to collect data on causes of death among children under age 5, (5) the community questionnaire, and (6) the fieldworker Questionnaire.

This study mainly used data collected from the household questionnaire and women’s questionnaire. The household questionnaire listed all of the usual members of and visitors to the selected households. Basic information was collected on the characteristics of each person listed, including age, sex, marital status, education, current work status, birth registration, and individual possession of a mobile phone. The women’s questionnaire collected information from ever-married women aged 15–49. Women responded to questions on background characteristics (for example, age, education, religion, and media exposure), reproductive history, use and source of family planning methods, ANC, delivery, postnatal and newborn care and breastfeeding, child immunizations, infant feeding practices and illness, marriage and sexual activities, fertility preferences, husbands’ background characteristics, and women’s work.

The questionnaires used in the survey were approved by the institutional review boards (IRBs) of the International Classification of Functioning, Disability and Health (ICF) and the Bangladesh Medical Research Council (BMRC). Both IRBs and the BMRC approved the protocols before the commencement of data collection activities.

Fieldwork for the main survey was carried out by several interviewing teams, with each team consisting of one male supervisor, one female field editor, five female interviewers, two health technicians, and one logistics staffer. Field supervisors, editors, interviewers, and health technicians filled out a two-page self-administered questionnaire on their general background characteristics. The ICF distributed and collected the questionnaires before the fieldworkers entered the field. No personal identifiers were attached to the BDHS fieldworkers’ data files. Data collection occurred in five phases, each about 4 weeks in duration. Data collection started on 24 October 2017 and was completed on 15 March 2018. The questionnaires were adapted to the local context and culture and translated into Bengali, the most commonly spoken language in Bangladesh. The questionnaires were pretested in 100 households. Based on observations in the field and suggestions made by the pretest teams, revisions were made.

Before the questionnaires were administered, informed consent was obtained from the mother. Efforts were made to maintain the privacy of respondents during interviews.

### 2.5. Statistical Analysis

The statistical programming language R was used for data cleaning, analysis, and visualization. Both descriptive statistics and inferential statistics were carried out in the study. Firstly, descriptive analyses like mean (SD), frequency, and percentage were performed for all the variables. Secondly, the chi-square (χ2) test was used to measure the association between PT and the explanatory variables. Finally, multiple logistic regression and crude and adjusted odds ratios were used to determine the factors of PT of the women in the study. In all analyses, the significance level was set at *p* < 0.05 (2-tailed) with a 95% confidence interval.

### 2.6. Ethical Consideration

This study used nationally representative BDHS 2017–2018 data, which are publicly available on the Demographic and Health Surveys (DHS) Program website: https://www.dhsprogram.com/data/dataset/Bangladesh_Standard-DHS_2017.cfm?flag=0 (accessed on 17 December 2021). The Office of Research Compliance Macro Institutional Review Board (Calverton, MD, USA) approved the survey, and the protocol was reviewed and approved by the National Ethics Review Committee of the Bangladesh Ministry of Health and Family Welfare. Informed consent was obtained from all the participants for the survey. Informed consent was obtained from parents/guardians of participants under 18 years of age. Since the data are secondary and thus allowed to be used for research, no additional ethical approval was needed for the authors of this study.

## 3. Results

### 3.1. Demographic Profile of Women

Frequency and percentage (weighted) distributions for the demographic variables of the study participants are shown in Table 1. The mean age of the women was 25.79 years, and approximately half (47.1%) of them were between 15 and 24 years old, followed by 44.8% who were between 25 and 34 years old. Nearly two-thirds (65.1%) of study participants lived in the rural areas of Bangladesh. It was also found that the majority (17%) of the women were from Chittagong, the southeastern part of Bangladesh, and the lowest proportion (10.3%) were from the Khulna and Barisal (10.3%) divisions, respectively. Although almost half (47%) of the total women had completed their secondary level of education, only 16.6% had completed higher study, and 7.3% had not received any formal education in their life. The study outcome also showed that more than ninety percent (91.5%) of respondents were Muslims, and the rest (9.5%) were from other religions, such as Hinduism, Christianity, or Buddhism. Less than 50% of the total participants were from a lower economic group, whereas two-fifths of them were from an upper economic group in society.

It is evident from the study that a maximum (59.3%) of women were housewives, and only 40.7% were currently engaged in a formal job (that is, they spent at least 8 h/day at an office). The average age of marriage was found to be 16.52 years, among wich 69.7% of women were married before reaching their 18th birthday, which is considered early marriage. We also found that slightly less than 50% of women received less than four antenatal care visits during their pregnancy period, while 8.1% did not receive any ANC. Two-thirds of the total survey respondents (67.8%) used any form of family planning during their reproductive age, and 62% of women had their own mobile phone.

### 3.2. Prevalence of Pregnancy Termination

**Figure 1:**
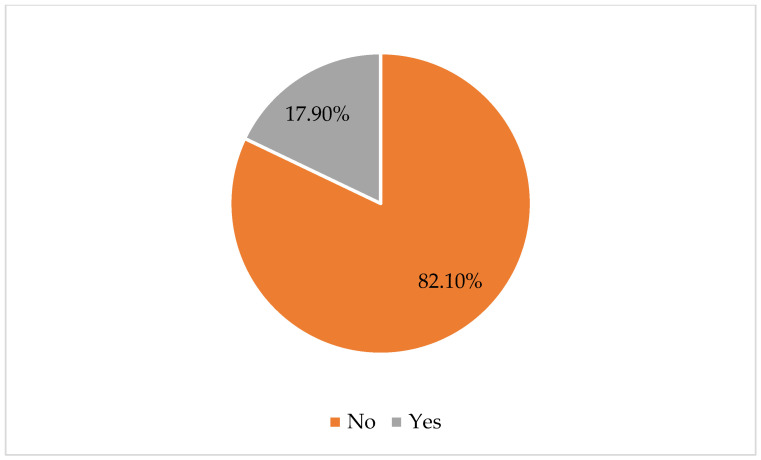
Pregnancy termination among the respondents.

This study indicated that approximately 18% of the surveyed women had experienced pregnancy termination (Figure 1).

This signifies that a considerable proportion of pregnant women encountered situations that prompted such decisions.

### 3.3. Association of Pregnancy Termination with the Explanatory Variables

The results revealed that PT was significantly (*p* < 0.001) higher among older women (36.4%); urban areas (20.3%) (*p* < 0.001); the residents of Dhaka (20%), Khulna (21%), and Sylhet divisions (20.5%) (*p* < 0.001); illiterate women (20.9%) (*p* < 0.001); Muslims (18.3%) (*p* = 0.005); job holders (19.4%) (*p* < 0.001); and women who possess their own mobile phone (18.9%) (*p* = 0.003) (Table 2).

In addition, more PT occurred among upper-economic-class women (19.7%), women who married at a relatively later age (>22 years) (18.2%), and women who completed four and above ANC visits to healthcare providers (18.1%), although these factors were not statistically significant (*p* ≥ 0.05).

### 3.4. Determinants of Pregnancy Termination

After adjusting all the variables in the logistics regression model, the result shows the determinants of PT, which are presented in Table 3. The probability of PT is more likely to increase with the age of the respondents. The age groups of 25–34, 35–44, and 45–49 years were 1.74 (AOR:1.74, *p* < 0.001), 2.70 (AOR:2.70, *p* < 0.001) and 3.49 (AOR:3.49, *p* = 0.004) times more likely to lose their pregnancy than the women aged 15–24 years old. Similarly, the likelihood of PT was substantially higher among the richest women (AOR:1.28, *p* = 0.027) and women who lived in the Sylhet division (AOR:1.28, *p* = 0.036). In addition, women with jobs were 15% more likely (AOR:1.15, *p* = 0.041) to have PT than those who did not have a job. Likewise, women who had their own mobile phones had 1.22 times higher odds (AOR:1.22, *p* = 0.002) of terminating their pregnancies than their counterparts.

On the other hand, rural women were less likely to have PT (AOR:0.81, *p* = 0.002) than the urban women, which indicates that the urban women, remarkably, had a 19% higher chance of ending their pregnancy than the women who lived in the countryside. The study also determined that the probability of PT decreased among more educated women (AOR:0.72, *p* = 0.027), non- Muslims (AOR:0.74, *p* = 0.010), and women who were married at a later age (AOR:0.71, *p* = 0.036).

## 4. Discussion

Identifying potential factors contributing to the elevated prevalence of PT is essential for healthcare practitioners and stakeholders to prioritize interventions targeting a reduction in PT and maternal fatalities. However, we found a lack of comprehensive research on this important reproductive health issue in Bangladesh. Therefore, this study was carried out based on the BDHS 2017–2018 data to determine the prevalence and factors of PT among reproductive-aged women in Bangladesh.

The results estimated that about one-fifth (18%) of the women in Bangladesh had terminated a pregnancy. Age, place of residence, level of education, religion, wealth index, working status, age at marriage, and having a personal mobile phone are the significant factors of PT in Bangladeshi women.

This study’s uniqueness lies in its exclusive examination of the prevalence and factors of PT in Bangladesh using the BDHS dataset, which is still a nationally representative latest dataset.

The prevalence of PT in Bangladesh is higher than the mean weighted prevalence (13%) of pregnancy termination in 36 other low- and middle-income countries [8]. On the contrary, Chinese women have a slightly higher prevalence of PT than Bangladeshi women [37]. Moreover, PT (in the form of miscarriage) declined significantly between 1998 and 2016 in Finland [38]. These discrepancies found in the prevalence of PT may be due to the variations in study locations, study populations, and study times.

Women’s age was determined to be one of the significant risk factors for PT, where it was observed that the chance of PT significantly increased with the age of the women. PT was found to be highly prevalent among older women compared to younger women. The findings of this study are consistent with the findings of some prior studies [39,40,41]. This positive association could be attributed to the fact that pregnancy-related complications like pre-eclampsia, ectopic pregnancy, and gestational diabetes mellitus, which may lead to miscarriages or stillbirths, increase with the age [42,43,44]. In addition, older women may already have their expected family size, resulting in them ending new pregnancies.

The current study depicted that women living in rural areas have a lower chance of PT than urban women, which is supported by some other previous studies [8,39]. Another study found similar results, stating that urban women have higher abortion rates than rural women [45]. A possible reason for this is that the women living in rural regions cannot afford to terminate their pregnancy [46,47]. On the contrary, this result is not endorsed by the study of Tamang A. et al., who found that PT was more frequent among rural women. A plausible reason for this difference is the different geographic locations, variation in the characteristics of the study population, and different times of these studies [48].

This study found geographical variation in PT in Bangladesh between administrative divisions. After adjusting covariates, this study determined a higher prevalence of PT in the Sylhet division, in the northeastern part of the country, and the Khulna division, in the southwestern part of the country. On the other hand, Chittagong, the southeastern part of the country, has the lowest probability of PT in Bangladesh. A possible explanation for this is considerable socio-economic factors, distance to facilities, and uneven distribution of public health resources in these regions.

This study’s results show that the probability of PT is significantly lower among the more educated women, which is consistent with previous studies conducted in Ethiopia [49,50] and elsewhere [51,52,53]. This could be attributed to the fact that educated women are effectively preventing unwanted pregnancies by utilizing contemporary contraceptive techniques. Moreover, less educated women are less likely to use contraceptive methods; thereby, they are more likely to have unintentional pregnancies, resulting in induced abortion. However, this study’s results contradict the findings of some other studies, which found that more educated women have a higher chance of PT than less educated women [54,55,56,57,58,59]. The differences in the sociodemographic characteristics of the study populations and study settings may be a possible explanation for such variation.

It was also observed that Muslim women were more likely to terminate a pregnancy than women of other religions like Hinduism, Christianity, and Buddhism. This is contradictory to the findings of Klutsey [60] and Ahiadeke [61], who found the prevalence of abortion was high among Christians. As most of the population of Bangladesh are Muslims, abortion is prohibited in the country [32]. However, PT can occur through various procedures like miscarriage, stillbirth, MR, etc., which may be more common among Muslim women.

Economic status has been found to be a significant contributor to PT. More affluent women have a higher likelihood of PT than the poorest women. This study finding is corroborated by previous studies conducted in Nepal, Ghana, India, and Nigeria [23,39,54,55,62,63,64]. In addition, some studies confirm that the odds of PT are higher among upper-economic-class women [51,54,56,65,66,67]. However, some studies in America and Brazil have determined that PT is higher among women in the lower socioeconomic classes. A possible reason for these mixed findings may be the ability of solvent women in Bangladesh to afford the cost of PT, although intentional abortion is illegal in the country, except for medical reasons concerning the mother [56].

There is a significant difference in PT between housewives and working women in Bangladesh. Women who have a formal job [i.e., who spent at least 8 h/day at an office] have a higher chance of PT, which aligns with the previous study of Ahinkorah et al. [40]. Another study among Ghanaian women using a DHS dataset from Ghana yielded similar results. The decision-making power of the women, prioritizing a career over having a baby, and being more aware of contraception (including MR) might be the reasons that working women terminate their pregnancy [56,68]. Some previous studies have also revealed that work itself may cause spontaneous abortion [69,70,71]. For example, a study in Nepal found that the women who are involved in agricultural activity have high stillbirth rates [72]. Therefore, this study emphasizes that pregnant women should receive sufficient support from their work environment.

The chance of termination of pregnancy reduced with the advanced age of marriage of the women. This study determined that women who were married before 18 years had a higher likelihood of ending their pregnancy. Similar findings were also observed in some other studies [59,73]. A possible explanation for this may be that early marriage may cause early pregnancy, which increases the risk of eclampsia, puerperal endometritis, and systemic infections than women who become pregnant at 20–24 years. Consequently, babies of adolescent mothers are at higher risks of low birth weight, preterm birth, and severe neonatal conditions. Therefore, women who become pregnant during this adolescent period may wish to avoid risk, and they may terminate their pregnancy instead of continuing it, even if they may have a miscarriage or stillbirth [74].

PT was found to be higher among mobile phone users in Bangladeshi women. One possible explanation for this is that mobile phones, as a communication technology, can increase exposure to mass media—such as television, radio, etc.—among women, enabling them to make their own decisions by providing sufficient information [39].

### 4.1. Strengths and Limitations

The current study solely assessed the prevalence of PT utilizing the BDHS dataset, which remains the most recent publicly accessible data in the country. This study used a large number of samples, which enhanced the generalizability of the results at a national level.

This study has some limitations. Firstly, the study was subject to recall bias as the respondents had to recall previous events, such as their pregnancy termination history, age of marriage, contraceptive use, etc. In addition, the BDHS data do not specifically classify the type of PT. Therefore, it was difficult to classify whether PTs were spontaneous or induced. Thus, this study could not differentiate circumstances that may have led to miscarriage compared with induced termination. Moreover, this study could not identify a causal association between PT and the explanatory variables due to its cross-sectional nature. Furthermore, BDHS 2017–2018 data were used, which are not the most recent set. We had to use this dataset as it is the only available latest nationally representative dataset encompassing the variables used. Lastly, there were some other relevant explanatory variables like gender issues, violence, medical complications, and extramarital affairs that could not be considered during data collection in this study.

### 4.2. Message to Policy Makers

This study provides some key points to be considered by policy makers. Firstly, maternal healthcare services should be made more accessible and available, especially for older women. Secondly, the working environment should be safe for pregnant women. Women should be encouraged to have their baby early in their conjugal life.

## 5. Conclusions

Women of advanced age, of upper socio-economic class, who have a job, and who own a mobile phone have a higher likelihood of terminating their pregnancy. Meanwhile, PT was lower among more educated women, rural residents, non- Muslims, and women who married later in life. Public health stakeholders and policy makers should account for these factors when creating interventions to reduce PT among Bangladeshi women. These results can facilitate further epidemiological research to elucidate the underlying causes of PT across diverse rural and urban environments and among various socio-demographic cohorts in Bangladesh. Future studies should consider the distinction between spontaneous abortion, induced abortion, and stillbirths so that the actual PT scenario can be factored in.

## Figures and Tables

**Table 1:** Socio-demographic characteristics of the participants (N = 8759).

Characteristics	Frequency (n)	Percent (%)
**Age group in years**		
**(Mean SD) years**	25.79 (5.68)	
15–24	4128	47.1
25–34	3920	44.8
35–44	689	7.9
45–49	22	0.3
**Place of residence**		
Urban	3057	34.9
Rural	5702	65.1
**Divisions**		
Barisal	906	10.3
Chittagong	1446	16.5
Dhaka	1304	14.9
Khulna	904	10.3
Mymensingh	1025	11.7
Rajshahi	912	10.4
Rangpur	971	11.1
Sylhet	1291	14.7
**Level of education**		
No education	642	7.3
Primary	2548	29.1
Secondary	4115	47.0
Higher	1454	16.6
**Religion**		
Islam	8018	91.5
Other	741	8.5
**Wealth quintile**		
Poorest	1928	22.0
Poorer	1755	20.0
Middle	1563	17.8
Richer	1737	19.8
Richest	1776	20.3
**Respondent’s current employment**		
Housewife	5195	59.3
Job holder	3564	40.7
**Age at marriage in years**		
**(Mean SD) years**	16.52 (2.86)	
<18	6103	69.7
18–22	2305	26.3
>22	351	4.0
**Antenatal visits**		
No visits	408	8.1
1–3 times	2190	43.7
≥4	2414	48.2
**Contraceptive use**		
No contraceptive use	2822	32.2
Contraceptive use	5937	67.8
**Mobile phone possession**		
No	3308	37.8
Yes	5451	62.2

**Table 2:** Distribution of the characteristics of the women by PT.

Characteristics	Pregnancy Termination		*p* Value *
	No n (%)	Yes n (%)	
**Age group in years**			
15–24	3587 (86.9)	541 (13.1)	<0.001
25–34	3099 (79.1)	821 (20.9)	
35–44	488 (70.8)	201 (29.2)	
45–49	14 (63.6)	8 (36.4)	
**Place of residence**			
Urban	2435 (79.7)	622 (20.3)	<0.001
Rural	4753 (83.4)	949 (16.6)	
**Divisions**			
Barisal	754 (83.2)	152 (16.8)	<0.001
Chittagong	1262 (87.3)	183 (12.6)	
Dhaka	1043 (80.0)	261 (20.0)	
Khulna	715 (79.1)	190 (21.0)	
Mymensingh	852 (83.1)	173 (16.9)	
Rajshahi	753 (82.6)	159 (17.4)	
Rangpur	783 (80.6)	188 (19.4)	
Sylhet	1026 (79.5)	265 (20.5)	
**Level of education**			
No education	508 (79.1)	134 (20.9)	<0.001
Primary	2030 (79.7)	518 (20.3)	
Secondary	3422 (83.2)	693 (16.8)	
Higher	1228 (84.5)	226 (15.5)	
**Religion**			
Muslim	6552 (81.7)	1466 (18.3)	0.005
Other	636 (85.8)	105 (14.2)	
**Wealth quintile**			
Poorest	1608 (83.4)	320 (16.6)	0.141
Poorer	1451 (82.7)	304 (17.3)	
Middle	1286 (82.3)	277 (17.7)	
Richer	1417 (81.6)	320 (18.4)	
Richest	1426 (80.3)	350 (19.7)	
**Respondent’s current employment**			
Housewife	4314 (83.0)	881 (17.0)	<0.001
Job holder	2874 (80.6)	690 (19.4)	
**Age at marriage in years**			
<18	5000 (81.9)	1103 (18.1)	0.835
18–22	1901 (82.5)	404 (17.5)	
>22	287 (81.8)	64 (18.2)	
**Antenatal visits**			
No visits	346 (84.8)	62 (15.2)	0.073
1–3 times	1843 (84.2)	347 (15.8)	
≥4	1976 (81.9)	438 (18.1)	
**Contraceptive use**			
No contraceptive use	2316 (82.1)	506 (17.9)	0.993
Contraceptive use	4872 (82.1)	1065 (17.9)	
**Mobile phone possession**			
No	2767 (83.6)	541 (16.4)	0.003
Yes	4421 (81.1)	1030 (18.9)	

* Pearson’s chi-square test was performed on the categorical variables; statistical significance was declared at *p* < 0.05.

**Table 3:** Factors associated with PT among reproductive-aged women in Bangladesh.

Factors	COR **	CI (95%)		*p* Value	AOR **	CI (95%)		*p* Value
		Lower Limit	Upper Limit			Lower Limit	Upper Limit	
**Age group (years)**								
15–24 (ref)	1.00	--	--		1.00	--	--	
25–34	1.75	1.56	1.98	<0.001	1.74	1.54	1.98	<0.001
35–44	2.73	2.26	3.29	<0.001	2.70	2.20	3.30	<0.001
45–49	3.79	1.58	9.07	0.003	3.49	1.42	8.54	0.004
**Place of residence**								
Urban (ref)	1.00	--	--		1.00	--	--	
Rural	0.78	0.69	0.87	<0.001	0.81	0.71	0.92	0.002
**Divisions**								
Barisal (ref)	1.00	--	--		1.00	--	--	
Chittagong	0.72	0.57	0.91	0.006	0.69	0.54	0.88	0.003
Dhaka	1.24	0.99	1.54	0.055	1.10	0.87	1.39	0.402
Khulna	1.31	1.03	1.66	0.025	1.31	1.03	1.68	0.027
Mymensingh	1.00	0.79	1.27	0.953	1.01	0.79	1.29	0.883
Rajshahi	1.04	0.82	1.33	0.710	1.05	0.81	1.34	0.693
Rangpur	1.19	0.94	1.50	0.147	1.23	0.96	1.56	0.090
Sylhet	1.28	1.02	1.59	0.028	1.28	1.01	1.61	0.036
**Level of education**								
No education (ref)	1.00	--	--		1.00	--	--	
Primary	0.96	0.78	1.20	0.761	1.10	0.88	1.38	0.381
Secondary	0.76	0.62	0.94	0.012	0.90	0.71	1.13	0.382
Higher	0.69	0.55	0.88	0.003	0.72	0.54	0.96	0.027
**Religion**								
Muslim (ref)	1.00	--	--	--	1.00	--	--	
Other	0.74	0.60	0.91	0.005	0.74	0.59	0.93	0.010
**Wealth quintile**								
Poorest (ref)	1.00	--	--		1.00	--	--	
Poorer	0.81	0.68	0.95	0.014	1.10	0.92	1.31	0.277
Middle	0.85	0.72	1.01	0.068	1.15	0.95	1.39	0.135
Richer	0.87	0.73	1.04	0.143	1.17	0.96	1.42	0.101
Richest	0.92	0.77	1.08	0.333	1.28	1.02	1.60	0.027
**Respondent’s current employment**								
Housewife (ref)	1.00	--	--		1.00	--	--	
Job holder	1.18	1.05	1.31	0.004	1.15	1.02	1.39	0.041
**Age at marriage in years**								
<18 (ref)	1.00	--	--		1.00	--	--	
18–22	0.96	0.85	1.09	0.560	0.88	0.76	1.01	0.080
>22	1.01	0.76	1.33	0.939	0.71	0.52	0.97	0.036
**Contraceptive use**								
No contraceptive use (ref)	1.00	--	--		1.00	--	--	
Contraceptive use	0.99	0.88	1.12	0.993	1.09	0.96	1.23	0.159
**Mobile phone possession**								
No (ref)	1.00	--	--		1.00	--	--	
Yes	1.19	1.06	1.34	0.003	1.22	1.07	1.38	0.002

** Multiple logistic regression was performed. (ref): Reference category. Adjusted for all the variables presented in the table. Statistically significant at *p* < 0.05. COR: crude odds ratio. AOR: adjusted odds ratio.

## Data Availability

This study used publicly available secondary data.
